# Neutrophil Extracellular Traps Activate Proinflammatory Functions of Human Neutrophils

**DOI:** 10.3389/fimmu.2021.636954

**Published:** 2021-06-08

**Authors:** Daniel Dömer, Tabea Walther, Sonja Möller, Martina Behnen, Tamás Laskay

**Affiliations:** Department of Infectious Diseases and Microbiology, University of Lübeck, Lübeck, Germany

**Keywords:** neutrophil extracellular traps, neutrophils, inflammation, effector functions, NET formation, ROS production, BAFF

## Abstract

Neutrophil extracellular traps (NETs) consist of decondensed nuclear chromatin that is associated with proteins and are released by neutrophils during an inflammatory response. Released NETs are able to capture pathogens, prevent their dissemination and potentially kill them *via* antimicrobial peptides and proteins that are associated with the decondensed chromatin. In addition to their antimicrobial functions, NETs have also been shown to exert immunomodulatory effects by activation and differentiation of macrophages, dendritic cells and T cells. However, the effect of NETs on neutrophil functions is poorly understood. Here we report the first comprehensive study regarding the effects of NETs on human primary neutrophils *in vitro*. NETs were isolated from cultures of PMA-exposed neutrophils. Exposure of neutrophils to isolated NETs resulted in the activation of several neutrophil functions in a concentration-dependent manner. NETs induced exocytosis of granules, the production of reactive oxygen species (ROS) by the NADPH oxidase NOX2, NOX2-dependent NET formation, increased the phagocytosis and killing of microbial pathogens. Furthermore, NETs induced the secretion of the proinflammatory chemokine IL-8 and the B-cell-activating cytokine BAFF. We could show that the NET-induced activation of neutrophils occurs by pathways that involve the phosphorylation of Akt, ERK1/2 and p38. Taken together our results provide further insights into the proinflammatory role of NETs by activating neutrophil effector function and further supports the view that NETs can amplify inflammatory events. On the one hand the amplified functions enhance the antimicrobial defense. On the other hand, NET-amplified neutrophil functions can be involved in the pathophysiology of NET-associated diseases. In addition, NETs can connect the innate and adaptive immune system by inducing the secretion of the B-cell-activating cytokine BAFF.

## Introduction

Neutrophils are part of the first line of defense against invading pathogens and are rapidly recruited to sites of tissue damage and infection. Equipped with highly potent antimicrobial effector functions, neutrophils can kill a wide array of microbial pathogens. These effector functions can be divided in intra- and extracellular responses [reviewed in (1)]. During the intracellular antimicrobial response, neutrophils phagocytose a pathogen. In the phagolysosome the pathogen is exposed to antimicrobial peptides, proteases, and reactive oxygen species (ROS) that originate from the NADPH-oxidase NOX2 (2). During the extracellular response, exocytosis of granules releases the antimicrobial granular content and NOX2-derived ROS into the extracellular environment to kill and prevent the dissemination of an invading pathogen. Furthermore, neutrophil extracellular traps (NETs) are released, which are able to capture and kill the invading pathogen (3). NETs are fibrous web-like structures that are composed of de-condensed chromatin, decorated with anti-microbial granule components, including neutrophil elastase (NE) and myeloperoxidase (MPO) (4). The underlying mechanisms of NET formation are not yet completely understood. However, it is widely accepted that during NET formation nuclear chromatin is de-condensed, loaded with granular proteins and finally released into the extracellular space (5). Since the first report of NET formation induced by bacteria (3), several other stimuli have been reported to induce NET formation including viruses (6), cholesterol and urate crystals (7), cytokines (8), calcium ionophores (9), bacterial lipopolysaccharides (10) and phorbol-12-myristat-13-acetat (PMA) (11). Albeit the exact process of NET formation differs dependent on the stimulus used NET-inducing stimuli can be divided in NOX2-dependent and NOX2-independent stimuli (5). While NOX2-dependent stimuli require the formation of ROS by NOX2, NOX2-independent stimuli require mitochondrial-derived ROS (12, 13). Furthermore, NOX2-independent NET formation requires citrullination of Histone H3 for decondensation of chromatin during NET formation by the peptidyl arginine deiminase 4 (PAD4) (8, 14). However, due to conflicting reports, the requirement of PAD4 activity for NET formation is controversial (8, 9, 14, 15).

Albeit the release of NETs is crucial for an effective defense against pathogens, uncontrolled release of NETs is associated with the pathologies of chronic inflammation and auto-immune diseases (12). The excessive release of NETs can cause tissue damage and initiate inflammation. The tissue damage, at least in part, is associated with direct killing of cells as shown in cultures of epithelial and endothelial cells (16–19). In addition, exposure of epithelial cells to NETs was reported to lead to the release of the proinflammatory chemokine IL-8 (18, 19).

An increasing number of studies report that NETs promote inflammation by interacting with several cells of the immune system. NETs were reported to induce the production of type I IFN by plasmacytoid dendritic cells (pDC) (20). Exposure of macrophages to NETs facilitated the release of IL-1β and IL-18 in a process that involves activation of the NLRP3 inflammasome (7, 21) and also directly induced the secretion of other proinflammatory cytokines including IL-8, IL-6 and TNFα (22, 23). Interestingly, NETs exert also anti-inflammatory effects on macrophages and dendritic cells by decreasing LPS-induced secretion of proinflammatory cytokines (22, 24) and the expression of antigen-presenting molecules by dendritic cells (24). Furthermore, exposure to NETs was reported to reduce the expression of IL-4R by monocytes resulting in the reduced differentiation of anti-inflammatory macrophages (25). In addition, NETs were shown to promote polarization of macrophages towards a reparative phenotype (26). Mediated by an effect on monocytes, NETs were also reported to induce the differentiation of Th17 cells in cultures of PBMCs stimulated with anti-CD3/CD28 (27). In addition to such indirect effects, NETs can also directly modulate T cell functions, as shown by the NET-induced enhanced expression of CD69 and CD25 resulting in the priming of CD4^+^ T-cells (28). Thus, NETs are not only part of the antimicrobial response launched by neutrophils, but have also the potency to shape an inflammatory as well as adaptive immune response.

Little is known about the effect of NETs on neutrophils. Since neutrophils are the most abundant cells recruited to sites of ongoing inflammation, they are exposed to the microenvironment of NET-releasing neutrophils. Based on the reported proinflammatory effects of NETs on other cells types of the immune system we hypothesized that exposure of the newly arrived neutrophils to NETs affects their function and enhance their proinflammatory functions. Indeed, recent studies showed that NETs can induce the secretion of IL-8 and induce NET-formation of resting neutrophils (22, 29, 30). In the present study we investigated the effects of NETs on primary human neutrophils in a more comprehensive manner. We show that exposure to NETs leads to the surface expression of activation markers, enhances exocytosis, induce the formation of ROS and ROS-dependent NET formation, activates phagocytosis and the secretion of IL-8 and the B-cell-activating cytokine BAFF. Furthermore, we provide first insights into the involved pathways during the NET-induced activation of neutrophils.

## Materials and Methods

### Ethics Statement

Blood collection was conducted with the agreement and written consent form of each participant and was approved by the Ethical Committee of the University of Lübeck (20-097).

### Isolation of Primary Human Neutrophils

Peripheral blood was collected by venipuncture from healthy adult volunteers using lithium–heparin collection tubes (S-Monovette^®^ R 9 ml LH, Sarstedt, Nümbrecht, Germany). Blood was layered on a two-layer density gradient consisting of an upper layer of Histopaque 1077 (Sigma Aldrich, Steinheim, Germany) and a lower layer of Histopaque 1119 (Sigma Aldrich) and centrifuged for 5 min at 300 x g followed by 25 min at 800 x g. Cells from the upper layer consisting mainly of lymphocytes and monocytes were discarded. The granulocyte rich lower layer was collected, leaving the erythrocyte pellet at the bottom of the tube. Granulocytes were washed once in Dulbecco’s phosphate-buffered saline (DPBS) (Thermo Fisher, Grand Island, New York, USA) for 10 min at 800 x g, resuspended in complete medium [RPMI 1640 Medium (Sigma Aldrich)] supplemented with 2 mM L-glutamine (Merck, Darmstadt, Germany), 10 mM 4-(2-hydroxyethyl)-1-piperazineethanesulfonic acid (HEPES; Life Technologies, Darmstadt, Germany), 10% heat-inactivated FCS (Gibco, Germany), 100 U/ml penicillin, and 100 µg/ml streptomycin (Biochrom, Berlin, Germany) and further fractionated on a discontinuous Percoll^®^ (GE Healthcare, Braunschweig, Germany) gradient consisting of layers with densities of 1.105 g/ml (85%), 1.100 g/ml (80%), 1.087 g/ml (70%), and 1.081 g/ml (65%). After centrifugation for 25 min at 800 x g, the interface between the 80% and 70% Percoll^®^ layers was collected. The cells were washed once in DPBS for 10 min at 800 x g and resuspended in complete medium at a concentration of 5x10^6^ cells/ml. All described procedures were conducted at room temperature and under sterile conditions. FCS that was supplemented to the medium was heat-inactivated at 70°C instead of 56°C for all experiments that involve exposure of NETs to neutrophils. This was conducted to inactivate nucleases within the FCS that are still active after heat-inactivation at 56°C (31) Cell counting was conducted with a hemocytometer (Imp. Neubauer, 0.0025 mm^2^, depth 0.100 mm (VWR, Dresden, Germany) and crystal violet staining. The preparations contained >99% granulocytes, of which >96% were neutrophils and 1%–4% were eosinophils, as determined by Giemsa staining (Diff Quik^®^ Fix, Medion Diagnostics, Berlin, Germany) of cytocentrifuged (Shandon) samples (**Supplementary Figure 1A**).

### Induction and Isolation of NETs

Neutrophils were suspended in FCS-free medium [RPMI 1640 Medium (Sigma Aldrich)] supplemented with 2 mM L-glutamine (Merck), 10 mM HEPES (Life Technologies), 100 U/ml penicillin, and 100 µg/ml streptomycin (Biochrome). 6x10^6^ neutrophils (1x10^6^ cells/ml) were seeded per well in a 6-well-plate, stimulated with 20 nM PMA (Sigma-Aldrich) and incubated for 4 h at 37°C, 5% CO_2_. NETs were isolated as described (32), with some modification. After the incubation, neutrophils were washed twice by carefully removing the medium without disturbing the NETs on the well bottom and carefully layering of fresh medium. After the wash steps, released NETs and cell debris were collected in 700 µl of FCS-free medium. Cell debris and NET fragments were separated by centrifugation at 300 x g 10 minutes at 4°C. The NET-containing supernatant was transferred to a fresh tube without disrupting the pellet. NET-containing supernatants are from now on referred to as NETs. A portion of the isolated NETs was sterile filtered with a 0.2 µm filter (GE Healthcare Life Science) to remove the NET fragments. Filtered NETs are referred to as fNETs.

NET formation was monitored by SYTOX Green Kinetic assay (**Supplementary Figure 1B**) and fluorescence microscopy (**Supplementary Figure 1C**) in parallel cultures, to ensure that the material isolated from cultures of PMA-treated neutrophils are NETs. Isolated NETs were analyzed by using MPO/DNA ELISA and quantified by using the Quant-iT™ PicoGreen™ dsDNA Assay-Kit (Invitrogen™, Eugene, Oregon, USA) according to the manufacturer’s instructions.

### Digestion of NETs by DNase I

500 ng/ml NET DNA was incubated with 80 U/ml DNase I (New England Biolabs, Massachusetts, USA) for 2 h at 37°C. Successful digestion of NETs was analyzed by Pico green assay according to the manufacturer’s instructions.

### ROS Assays

The luminol-based chemiluminescence assay was used to detect the sum of intra- and extracellular ROS as described in (33). 4×10^5^ neutrophils (2x10^6^ cells/ml) were seeded per well in a 96-well LUMITRAC™ 600 plates (Greiner Bio-One, Frickenhausen Germany) and mixed with a final concentration of 60 µM luminol (Sigma-Aldrich). Neutrophils were exposed to 20 nM PMA, 200 ng/ml NETs, to corresponding filtered NETs or FCS-free medium alone. ROS-dependent luminol chemiluminescence was assessed using an infinite 200 reader and the Tecan i-control 1.7 Software (Tecan, Crailsheim, Germany). ROS release was monitored for 2 h every 2 min at 37°C, 5% CO_2_. For statistical analysis, the area under the curve (AUC) value of each sample was calculated. For inhibitor studies, neutrophils were treated with 10 µM GSK484, 2 µM DPI, 50 µM VAS2870 (all from Sigma-Aldrich) or left untreated for 30 min at 37°C, 5% CO_2_ prior to the addition of luminol and stimuli.

Extracellular superoxide was detected by using the lucigenin-amplified chemiluminescence assay. This assay was performed the same way as the luminol assay, but with 0.2 mM lucigenin (33) (Alexis Loerrach, Germany) instead of luminol.

### Analysis of Neutrophil Activation and Exocytosis by Flow Cytometry

To assess the activation of neutrophils by isolated NETs, the shedding of CD62L and expression of the indirect degranulation marker CD11b were analyzed by flow cytometry. 1x10^6^ neutrophils (5x10^6^ cells/ml) were incubated for 1 h at 37°C 5% CO_2_ in a 96-well plate in the presence of indicated stimuli. Afterwards, 5x10^5^ neutrophils were stained with FITC-conjugated mouse antibody (mAb) to human CD62L (BD) and PE-conjugated mAb to human CD11b (Dako Agilent, Santa Clara, USA). Analysis of exocytosis was conducted by measuring the expression of CD35, CD63 and CD66b using FITC-conjugated mAb to human CD35 (BioLegend, San Diego, USA), APC-conjugated mAb to human CD63 (BioLegend) and FITC-conjugated mAb to human CD66b (BD).

Staining of neutrophils was conducted as described (34). After the staining, cells were kept at 4°C and expression of the above listed markers was analyzed with a BD FACS Canto II (BD).

### Assessment of the Effect of NETs on NET Formation

To determine the induction of NET formation by NETs, neutrophils were exposed to 20 nM PMA, 200 ng/ml NETs, to corresponding filtered NETs or FCS-free medium alone for 4 h at 37°C, 5% CO_2_. Formation of NETs was monitored by SYTOX Green Kinetic assay, fluorescence microscopy and MPO/DNA ELISA.

### SYTOX Green Kinetic Assay

Kinetic analysis of NET formation was conducted using the cell impermeable dsDNA dye SYTOX™ Green Nucleic Acid Stain (Invitrogen™). 2x10^5^ neutrophils (1x10^6^ cells/ml) were seeded per well in a 96-well FLOUTRAC™ 600 plates (Greiner Bio-One) in FCS-free medium containing a final concentration of 5 µM SYTOX Green. NET formation was induced by addition of the mentioned stimuli, Sytox Green fluorescence was measured by using a plate reader and Tecan iControl software. NET formation was analyzed using Graph Pad Prism 6 software by determining the area under the curve (AUC) values of the SYTOX Green fluorescence over a time period of 4 h. For assays involving NET-induced NET formation, the SYTOX Green background signal was subtracted from values to compensate for the signal derived from added NETs. For inhibitor studies, neutrophils were treated with, 2 µM DPI, 50 µM VAS2870 (both from Sigma-Aldrich) or left untreated for 30 min at 37°C, 5% CO_2_ prior to the addition of SYTOX Green and stimuli.

### Fluorescence microscopy of NET formation

5x10^6^ neutrophils (1x10^6^ cells/ml) were seeded onto a poly-L-lysine-coated coverslip (Bedford, MA, USA) in FCS-free medium and NET formation was induced as described above. After 4 h, medium was aspirated and cells were fixed with 4% PFA for 30 min at 37°C, 5% CO_2_. Afterwards, neutrophils were rehydrated with ddH_2_O, stained with 100 nM SYTOX Green for 30 min and washed twice with DPBS. Finally, cover slides were sealed with prolong Gold antifade reagent (Invitrogen™) on a microscopy slide for 18 h at 4°C. Microscopy was conducted by Keyance BZ9000 microscope (Keyance, Osaka, Japan) using a 40x Plan Fluor EL NA0,60 objective (Nikon, Tokyo, Japan).

### MPO–DNA Complex (NET) ELISA

Since NETs contain both DNA and MPO, a MPO–DNA complex ELISA was used to detect and quantify soluble NETs in culture supernatants as described (35). Briefly, 96-well ELISA Maxisorp plates (Thermo Fisher) were coated with 5 μg/ml mouse anti-human MPO antibody (BioRad) over night at 4°C. After three washing steps and blocking with 1% BSA, 20 μl of cell culture supernatant (from 1×10^6^ cells/ml) together with 80 μl incubation buffer and 4 μl peroxidase labeled anti DNA monoclonal antibody (both from Cell Death Detection ELISA Plus, Roche) were added to the wells. Following 2 h incubation, the wells were washed once and 100 μl peroxidase substrate was added. The absorption at 405 nm was measured after 20 min in an ELISA reader (Tecan).

### Western Blot Analysis

Neutrophils (5×10^6^ cells/ml) were exposed to PMA, NETs, corresponding fNETs or FCS-free medium for 15 min at 37°C in FCS-free medium. Whole cell lysates were prepared as described (35). Western blot analysis was carried out by using antibodies against human phospho-Akt (Thr308), phospho-p44/42 MAPK (ERK1/2, thr202/Tyr204), phospho-p38 MAPK (Thr180/Tyr182), Akt, p44/p42 MAPK (ERK1/2), p38 MAPK or beta-actin (all from Cell Signaling Technology) and probed with HRP-conjugated anti-mouse IgG secondary antibody (New England Biolabs, USA). The signals were detected by using Immobilon Western Chemiluminescence HRP substrate (Millipore, USA) with a Fusion Fxt Chemiluminescence reader (Vilber Loumat, Germany). Signals of pAkt, pERK1/2, or pp38 were analyzed by densitometry using ImageJ software (NIH, USA). For the statistical analysis the measured densitometry values of proteins were corrected by the signal of the unphosphorylated proteins. Therefore, the signal of phospho proteins was divided by the signal of the respective unphosphorylated protein.

### Phagocytosis Assay

Neutrophils (5×10^5^ cells/100 μl) were preincubated for 30 min with PMA, NETs, fNETs or left untreated. Subsequently, FluoSphere carboxylate-modified latex microspheres (Invitrogen™) with a diameter of 1 μm at a final concentration of 0.015% (v/v) were added and the co-culture was incubated for further 30 min. Cultures were placed on ice to stop phagocytosis and cells were washed to remove non-ingested beads. A portion of cells from each condition was used for a Giemsa staining. Phagocytosis was assessed by flow cytometry using a BD FACS Canto II flow cytometer (BD) and by fluorescent microscopy of Giemsa-stained cytocentrifuge slides.

The phagocytosis of opsonized bacteria was investigated by using of *Staphylococcus aureus* and *Escherichia coli* Bioparticles. 100 µg of *Staphylococcus aureus* Bioparticle- Alexa Fluor™ 488 conjugated or *Escherichia coli* Bioparticle- Alexa Fluor™ 488 conjugated (both Invivogen, San Diego, USA) were opsonized using *S. aureus* Bioparticle opsonizing reagent or *E. coli* Bioparticle opsonizing reagent, respectively (both from Molecular Probes, Eugene, USA) for 1 h at 37°C. Afterwards, opsonized bioparticles were washed twice and suspended in PBS in a concentration of 33 µg/ml and kept on 4°C before adding them to neutrophils. Opsonized bioparticles where added to preincubated neutrophils (5×10^5^ cells/100 μl) at a 5 bioparticles to 1 neutrophil ratio. Incubation and analysis of phagocytosis by flow cytometry was conducted as described above for FluoSpheres.

### Assessment of Intracellular Killing of *Leishmania donovani* by Neutrophils

Cultivation of *L. donovani*, infection of neutrophils with *L. donovani* and assessment of killing of *L. donovani* was conducted as described (34). Briefly, infection was conducted by coincubation of neutrophils and *L. donovani* promastigotes in a 1:10 ratio for 3 h at 37°C. Successful infection was assessed by analyzing intracellular *L. donovani* by Gimsa-staining of cytocentrifuged samples. Infected neutrophils were washed by centrifugation to remove non-ingested *Leishmania* and suspended in fresh complete medium (5x10^6^ cells/ml). Infected neutrophils were exposed to NETs [50 ng/ml] or a corresponding dilution of fNETs for 18 h at 37°C. Complete medium served as negative control. IFNγ [200 U/ml] in combination with LPS [100 ng/ml], was used as positive control for the activation of neutrophil antimicrobial activity. Afterwards, a limited dilution assay was conducted by performing serial 1.5 fold dilutions of four replicates per condition in a 96-well flat-bottom plate. Survival of *L. donovani* was assessed after 10 days by analyzing the last dilution resulting in the growth of *L. donovani* in more than 50% of the wells.

### Cytokine Determination

Neutrophils (1x10^6^ cells/100 µl) were exposed to 100 U/ml IFNγ (R&D Systems, Wiesbaden, Germany) + 100 ng/ml LPS (Sigma-Aldrich), 1000 U/ml G-CSF (PeproTech, Hamburg, Germany), 100 ng/ml NETs, corresponding fNETs or complete medium for 21 h at 37°C, 5% CO_2_. Cultures were centrifuged at 300 x g for 10 minutes to separate cytokine-containing supernatants from cells. Detection of IL-8, IP-10, TNFα and BAFF in the supernatants was conducted by using Duo-Set ELISAs (R&D Systems) according to the manufacturer’s instructions. To correct for a potential presence of the analyzed cytokines in the NETs used for treatment of neutrophil cultures, the background signal of each stimulus alone was subtracted from the cytokine levels measured in the culture supernatants. Cytokine release was calculated using Graph Pad Prism 6 software by interpolation of unknown by a standard curve.

### Statistical Analysis

If not stated differently, the presented data were collected/generated from minimum of three independent experiments with neutrophils isolated from different blood donors. Statistical analysis was performed with the GraphPad Prism software 6 using the ordinary one-way ANOVA followed by Turkey’s multiple comparison test. A p-value ≤0.05 was considered statistically significant.

## Results

### NETs Activate Resting Neutrophils and Induce Exocytosis

**Figure 1:**
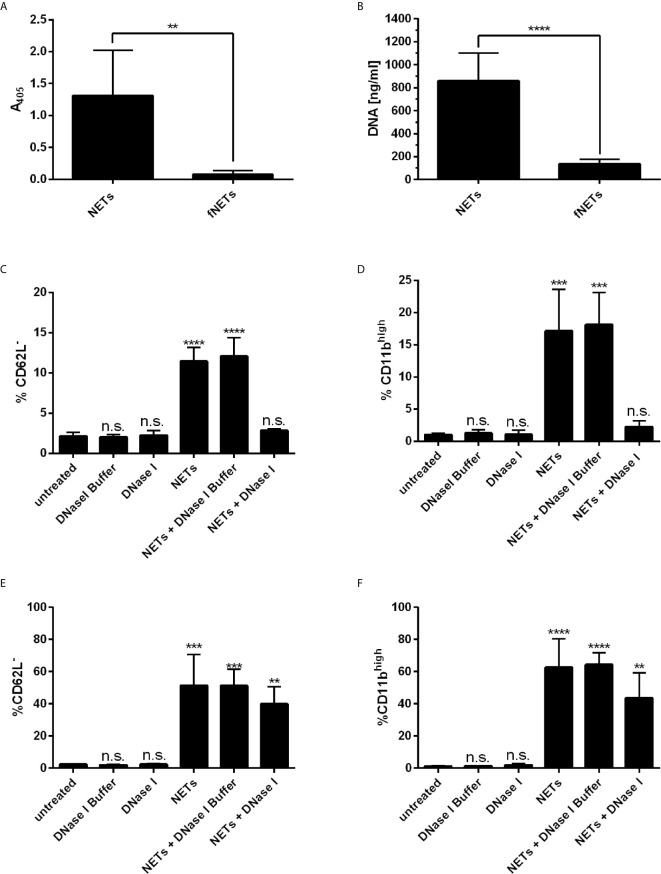
NETs activate human neutrophils. **(A, B)** NET-containing supernatants were filtered with a 0.2 µm filter to remove NETs. Removal of NETs was analyzed by detecting a MPO-DNA complex using a NET ELISA **(A)** and measureing DNA concentrations by Picogreen assay **(B)**. Statistical analysis by unpaired t-test. Asterisks indicate significant differences. n=7, **=p ≤ 0,01, ****=p ≤ 0,0001. **(C–F)** Neutrophils were incubated for 30 min **(C, D)** or 1 h **(E, F)** with NETs, NETs digested with DNase I or NETs incubated with DNase I buffer containinig no DNAse I. Neutrophil activation was assessed by analyzing the shedding of CD62L **(C, E)** and expression of CD11b **(D, F)**. Statistical analysis by ordinary one-way ANOVA with a *post hoc* Turkey’s test. Asterisks above the bars indicate significance compared to untreated cells. n=3, **=p ≤ 0,01, ***=p ≤ 0,001, ****=p ≤ 0,0001. n.s, not significant.

Recently, it has been reported that NETs activate neutrophils to express CD11b (22) and induce the shedding of CD62L (30). Thus, we tested whether our NETs are biologically active by analyzing the expression of these activation markers by flow cytometry. Exposing neutrophils to NETs induced the shedding of CD62L (**Supplementary Figure 2B**) and enhanced the expression of CD11b (**Supplementary Figure 2C**) indicating the biological activity of our NETs. The effects were concentration dependent (**Supplementary Figures 3A, B**). NETs at a concentration of 100 ng/ml NET-DNA or higher significantly induced the shedding of CD62L and enhanced expression of CD11b. To test, whether the stimulatory effect is due to soluble stimuli inside the NET-containing supernatants (e.g. remaining PMA from the isolation), we removed NETs by filtration and exposed cells to these filtered NETs (fNETs). To confirm that NETs were removed by the filtration, we analyzed our isolated NETs and the filtered NETs (fNETs) for a MPO-DNA complex by the NET ELISA and measured DNA content using Picogreen assay. NETs but not fNETs displayed a strong signal for a DNA-MPO complex (**Figure 1A**) and significant DNA concentrations (**Figure 1B**). This indicates, that our culture supernatants contain NETs, that can be excluded by filtration. Exposure of neutrophils to fNETs did not have an effect on CD62L and CD11b expression (**Supplementary Figures 2A, B**). Thus, activation of neutrophils is due to NETs and not remaining PMA or other soluble stimuli.

To further confirm that the effects on cell surface marker expression of neutrophils were indeed induced by NETs, we analyzed the activation of neutrophils by NETs that were digested with DNase I. Digestion of NETs significantly reduced the activation of neutrophils as measured after an exposure time of 30 min (**Figures 1C, D**). Interestingly, longer exposure of neutrophils to DNAse-I-degested NETs resulted in activation of neutrophils, albeit the level of activation was reduced compared to undigested NETs (**Figures 1E, F**). Measurement of the DNA content of DNase I-digested NETs revealed that low but measurable amounts of NET DNA were still present in NETs after DNase I digestion (**Supplementary Figure 2D**).

**Figure 2:**
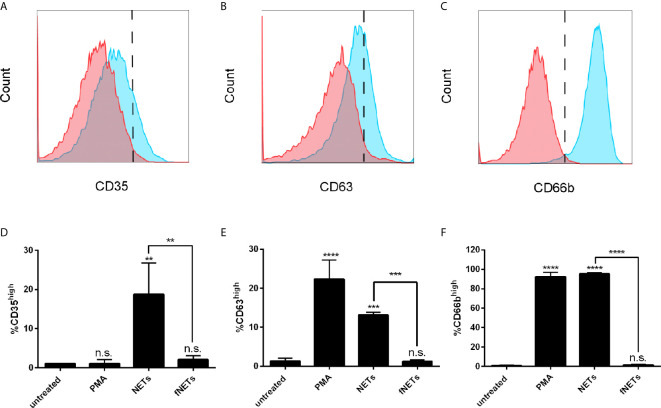
NETs induce the exocytosis of neutrophil granules. Neutrophils were exposed to PMA, NETs, fNETs or left untreated for 1 h and exocytosis was assessed by analyzing the expression of CD35 **(A, D)**, CD63 **(B, E)** and CD66b **(C, F)** by flow cytometry. **(A–C)** Representative histograms of neutrophils expressing CD35 **(A)**, CD63 **(B)** and CD66b **(C)**. Red=untreated neutrophils. Blue=NET-exposed neutrophils. Signals on the right side of the dashed line were considered as high expressing cells. **(D–F)** Statistical analysis of the expression of CD35 **(D)**, CD63 **(E)** and CD66b **(F)** by ordinary one-way ANOVA with a *post hoc* Turkey’s test. Asterisks above the bars indicate significance compared to untreated cells. n=3, **=p ≤ 0,01, ***=p ≤ 0,001, ****=p ≤ 0,0001. n.s, not significant.

Increased expression of CD11b by NET-exposed neutrophils suggests that NETs activate the exocytosis of granules. To test this, we analyzed the expression of the granule marker CD35 (secretory vesicles), CD63 (primary granules) and CD66b (secondary granules) of NET-exposed neutrophils by flow cytometry. Exposure of neutrophils to NETs induced an increased expression of CD35 (**Figures 2A, D**), CD63 (**Figures 2B, E**) and CD66b (**Figures 2C, F**), indicating that NETs induce the exocytosis of specific and azurophilic granules as well as secretory vesicles. Exposure to fNETs had no effect on neutrophils (**Figures 2D, E**).

In summary, NETs activate neutrophils and induce exocytosis. The activation of neutrophils by NETs was partially abrogated by digesting NETs with DNase I.

### NETs Activate ROS Production by Neutrophils

After having shown that isolated NETs exert an effect on the expression of neutrophil activation markers (**Figures 1A, B**), we addressed the question whether NETs activate antimicrobial effector functions of neutrophils. Since production of ROS by neutrophils is an important effector function to fight pathogens (2) we investigated if exposure to NETs increases ROS production by neutrophils. The luminol-based chemiluminescence assay was used to detect intra- and extracellular ROS species while the lucigenin-based luminescence assay was used to detect only extracellular ROS since lucigenin cannot penetrate the cell membrane (33).

**Figure 3:**
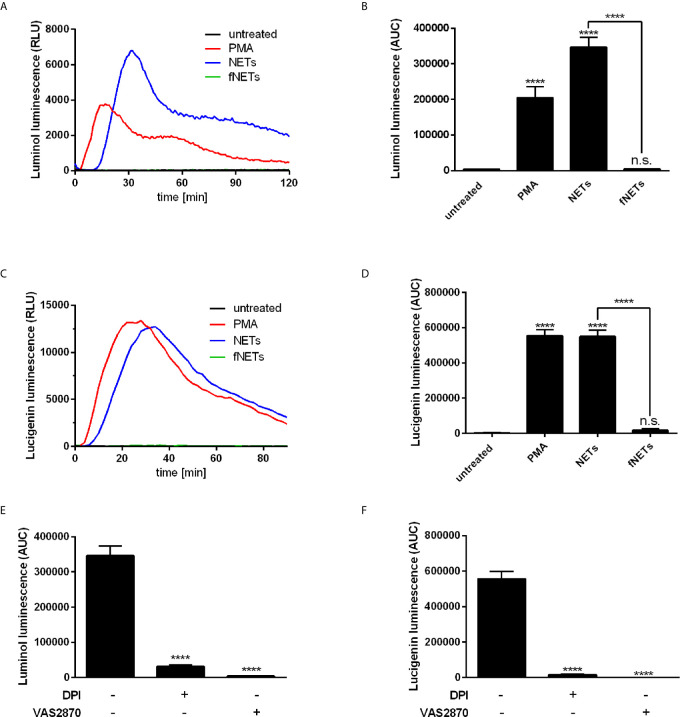
NETs induce ROS production by neutrophils. Neutrophils were labelled with luminol or lucigenin and treated with PMA, NETs, fNETs or left untreated. Production of total ROS was measured by luminol luminescence **(A, B, E)** and extracellular ROS by lucigenin luminescence **(C, D, F)**. **(A, C)** Representative kinetic curves of ROS production by neutrophils. **(B, D)** ROS production shown by calculating the area under the curve (AUC) values. **(E, F)** Neutrophils were treated with the inhibitors DPI, VAS2870 or left untreated for 30 minutes prior to addition of NETs. ROS production was assessed by using the luminol **(E)** or **l**ucigenin **(F)** assay. Statistical analysis by ordinary one-way ANOVA with a *post hoc* Turkey’s test. Asterisks above the bars indicate significance compared to untreated cells. n=3, ****=p ≤ 0,0001. n.s, not significant.

Exposure to NETs resulted in the formation of both intra- and extracellular ROS (**Figures 3A–D**). NETs induced ROS production at a similar or even higher level as PMA, which was used as positive control for the induction of ROS production (**Figures 3A–D**). Induction of ROS production occurred in a concentration-dependent manner (**Supplementary Figure 3C**). Exposure to fNETs did not induce neutrophil ROS production (**Figures 3A–D**).

To analyze the main source of ROS, we treated neutrophils with the inhibitors diphenyleneiodonioum (DPI) and VAS2870 before exposure to NETs. DPI inhibits both NOX2-derived ROS and ROS produced by mitochondrial respiration, whereas VAS2870 is a specific inhibitor of NOX2. Treatment of neutrophils with both DPI and VAS2870 resulted in the nearly complete abrogation of ROS production (**Figures 3E, F**) indicating that NETs activate NOX2 to produce ROS.

### NET-Induced NET Formation by Neutrophils Depends on NOX2 but Not PAD4

After having observed that NETs induce ROS production by neutrophils, we addressed the question whether NETs can induce the formation of further NETs, since ROS production is required for several NET-inducing stimuli (5).

**Figure 4:**
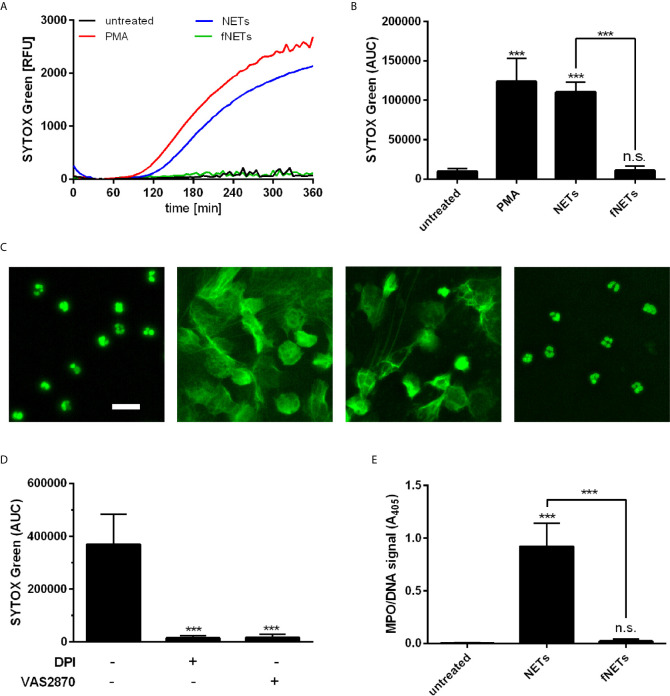
NETs induce NOX2-dependent NET formation. Neutrophils were incubated for 4 h with PMA, NETs, fNETs or left untreated. NET formation was analyzed by SYTOX green fluorescence **(A–C)** or MPO-DNA ELISA **(D)**. **(A)** Representative kinetic development of NET formation. **(B)** Statistical analysis of NET formation by calculating the area under the curve (AUC) values. **(C)** Fluorescence microscopy of SYTOX green stained neutrophils. Size bar= 20 µm. **(D)** Assessment of NET release by detection of a MPO-DNA complex by NET ELISA. **(E)** Neutrophils were treated with the inhibitors DPI, VAS2870, or left untreated for 30 minutes prior to exposure to NETs. NET formation was analyzed as described in **(B)**. Statistical analysis by ordinary one-way ANOVA with a *post hoc* Turkey’s test. Asterisks above the bars indicate significance compared to untreated cells. n=3, ***=p ≤ 0,001. n.s., not significant.

Neutrophils were exposed to NETs and NET formation was analyzed by SYTOX Green fluorescence and DNA/MPO ELISA. Exposure of neutrophils to NETs resulted in NET formation as assessed by detecting the release of DNA with SYTOX Green fluorescence (**Figures 4A, B**). The NET-induced NET formation showed similar time kinetics as seen after treatment with PMA (**Figure 4A**). The NET-induced formation of NETs was further confirmed by fluorescence microscopy (**Figure 4C**) and by quantification of DNA-bound MPO (**Figure 4E**). Exposure of neutrophils to fNETs did not lead to NET-formation (**Figures 4A–C, E**).

To further characterize the observed NET-induced NET formation in terms of ROS-dependence, neutrophils were pre-treated with the inhibitors DPI and VAS2870. Inhibition of ROS production by DPI and VAS2870 abrogated the NET formation upon NET exposure (**Figure 4D**). This indicates that NET-induced NET formation depends on NOX2-derived ROS production. Inhibition of PAD4 did not reduce NET formation by neutrophils exposed to NETs (data not shown).

In summary, these data show that NETs induce the formation of further NETs by neutrophils and that this NET-induced NET formation depends on NOX2.

### Phagocytic Activity of Neutrophils Is Enhanced by NETs

Next, we investigated whether NETs, in addition to their effects on ROS production and NET formation, also activate other antimicrobial effector functions of neutrophils. Since in a previous work, thymus-derived chromatin was shown to activate the phagocytic activity of neutrophils (36), we hypothesized that NETs also exert an activating effect on phagocytosis by neutrophils. Neutrophils were exposed to NETs and subsequently phagocytosis of fluorescence-labeled latex beads was analyzed by fluorescence microscopy and flow cytometry.

**Figure 5:**
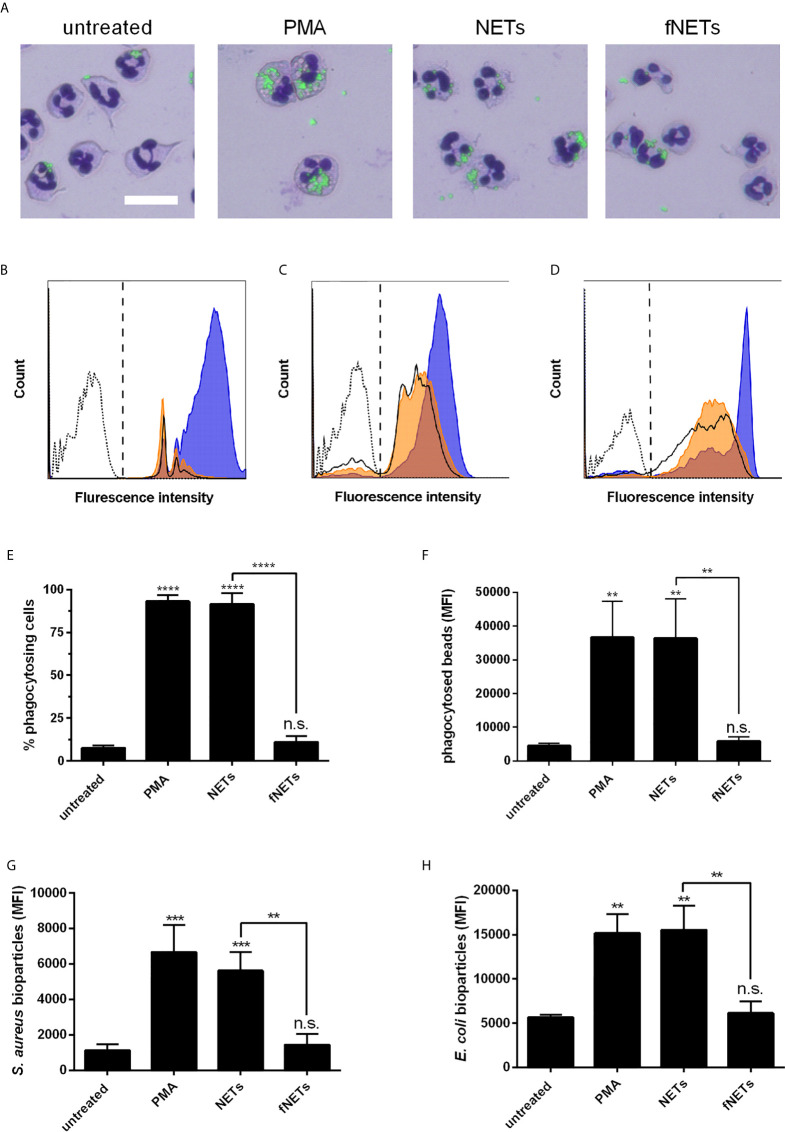
Activation of phagocytosis by NETs. Neutrophils were exposed to PMA, NETs, fNETs or left untreated and phagocytosis of fluorescent-labelled particles was assessed by microscopical examination and flow cytometry. **(A)** Phagocytosis of latex beads was visualized of Giemsa-stained neutrophils by fluorescence microscopy. Size bar=20 µm. **(B–D)** Representative histogram of neutrophils after phagocytosis of latex beads **(B)**, opsonized *S. aureus* bioparticles **(C)** or opsonized *E. coli* bioparticles **(D)**. Signals on the right side of the dashed line in panels B, C and D were considered as phagocytosing cells. Dotted line=neutrophils without particles; bold line=untreated neutrophils; orange=neutrophils exposed to fNETs; blue=neutrophils exposed to NETs. **(E–H)** Phagocytosis was assessed by analyzing the percent of neutrophils with ingested fluorescent beads **(E)** and mean fluorescence intensity (MFI) of cells that phagocytosed beads **(F)** *S. aureus* **(G)** or *E. coli* **(H)** by flow cytometry. Statistical analysis by ordinary one-way ANOVA with a *post hoc* Turkey’s test. Asterisks above the bars indicate significance compared to untreated cells. n=3 **=p ≤ 0,01, ***=p ≤ 0,001. n.s., not significant.

Exposure of neutrophils to NETs strongly enhanced the phagocytosis of latex beads by neutrophils (**Figures 5A, B**). Both the ratio of phagocytosing cells and the number of phagocytosed particles per neutrophil were increased upon exposure to NETs (**Figures 5E, F**). As observed for other neutrophil functions, the stimulation of phagocytosis by NETs was also concentration-dependent (**Supplementary Figures 2E, F**). fNETs did not affect neutrophil phagocytosis (**Figure 5**).

To test whether the observed increased phagocytosis by NET-exposed neutrophils could be beneficial regarding the defense against invading pathogens, we analyzed the phagocytosis of opsonized *Staphylococcus aureus* and *Escherichia coli* bioparticles. Exposure of neutrophils to NETs increased phagocytosis of opsonized *S. aureus* (**Figures 5C, G**) and *E. coli* bioparticles (**Figures 5D, H**). fNETs had no effect on the phagocytosis of either bacterium (**Figure 5**).

In summary, these data indicate that exposure to NETs results in enhanced phagocytic activity of neutrophils.

### Microbicidal Activity of Neutrophil Is Enhanced Upon NET Exposure

**Figure 6:**
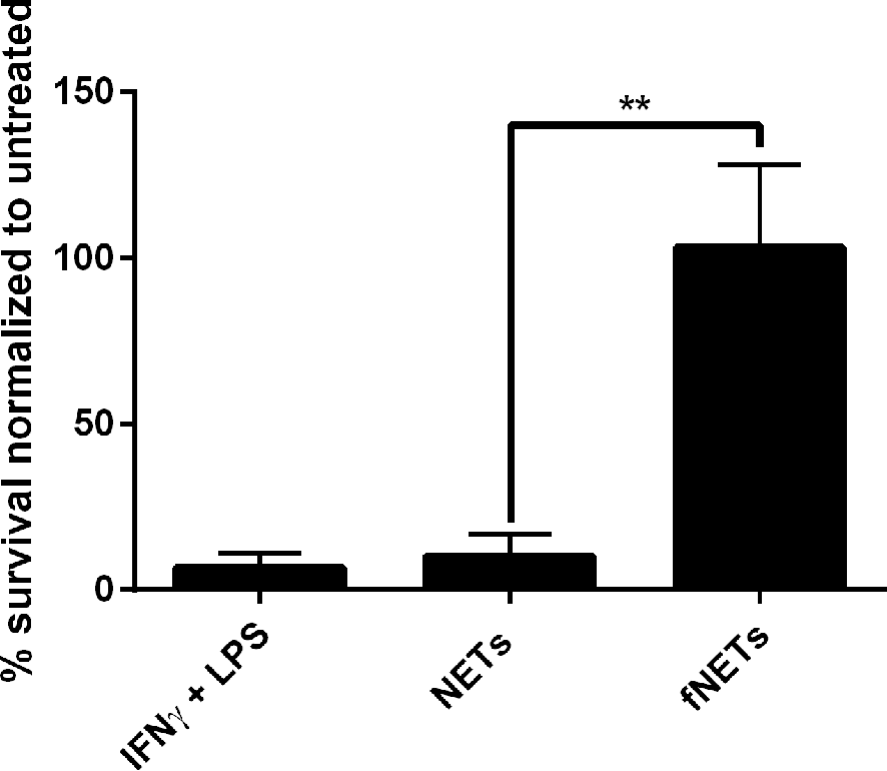
Exposure to NETs results in enhanced intracellular killing of *L. donovani* by neutrophils. Neutrophils were infected with *L. donovani* promastigotes and exposed to NETs, fNETs or a combination of IFNγ and LPS for 18 h. Survival of *Leishmania* was assessed by using a limited dilution assay calculating % of viable *Leishmania* compared to untreated neutrophils. Statistical analysis by unpaired t-test. n=3, **=p ≤ 0,01.

Our findings, that NET-exposure not only induces ROS production but also increases phagocytosis of pathogens by neutrophils suggests that NET-exposure increases antimicrobial activity of neutrophils. To test this, we exposed *Leishmania donovani*-infected neutrophils to NETs and analyzed intracellular killing of this pathogen. Exposure of neutrophils to NETs increased the killing of *L. donovani* (**Figure 6**), while exposure to fNETs did not affect intracellular killing. NETs alone did not kill *L. donovani* (data not shown) indicating that the increased killing of *L. donovani* is due to activation of neutrophils by NETs.

### NETs Induce the Secretion of IL-8 and BAFF by Neutrophils

In addition to their antimicrobial effector functions neutrophils can regulate inflammatory and immune responses by releasing cytokines (37). After having seen that NETs can activate effector functions of neutrophils we analyzed, whether NETs also influence the regulatory functions of neutrophils. Since NETs were shown to induce the release of proinflammatory cytokines by macrophages (7, 22), we assessed the secretion of the proinflammatory cytokine/chemokine TNFα, BAFF, CXCL8/IL-8 and CXCL10/IP-10 by primary human neutrophils upon exposure to NETs. Neutrophils were treated with NETs for 18 h and collected supernatants were analyzed by ELISA. G-CSF was used as positive control for the induction of BAFF production (38). IFNγ in combination with LPS was used as positive control for IP-10 production since simultaneous exposure of neutrophils to IFNγ and LPS was shown to induce the secretion of CXCL10/IP-10 by neutrophils (39).

**Figure 7:**
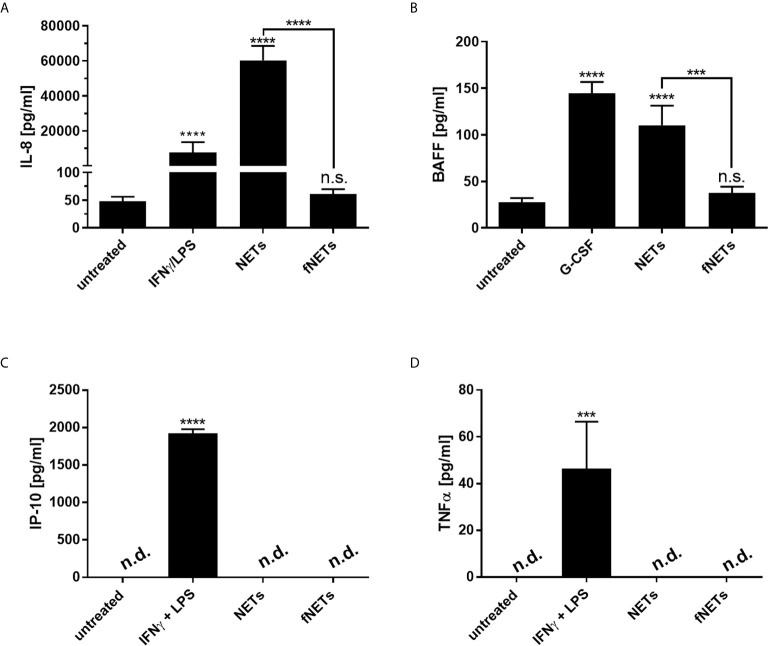
NET-induced cytokine secretion by neutrophils. Neutrophils were treated with NETs fNETs, IFNγ + LPS, G-CSF or left untreated for 18 h. **(A–D)** The amounts of IL-8, BAFF, IP-10 and TNFα were measured in the supernatants by ELISA. Cytokine concentrations were determined by interpolating the concentrations from a standard curve. Statistical analysis by ordinary one-way ANOVA with a *post hoc* Turkey’s test. Asterisks above the bars indicate significance compared to untreated cells. n=3 ***=p ≤ 0,001, ****=p ≤ 0,0001. n.s, not significant; n.d, not detected.

Exposure to NETs induced the secretion of IL-8 and BAFF by neutrophils (**Figures 7A, B**). However, NETs did not induce the secretion of CXCL10/IP-10 and TNFα (**Figures 7C, D**). No cytokine secretion could be observed after the exposure of neutrophils to fNETs (**Figure 7**).

Taken together, NETs affect the immunomodulatory functions of neutrophils by inducing the secretion of cytokines.

### Exposure of Neutrophils to NETs Leads to the Phosphorylation of Akt, ERK1/2 and p38

**Figure 8:**
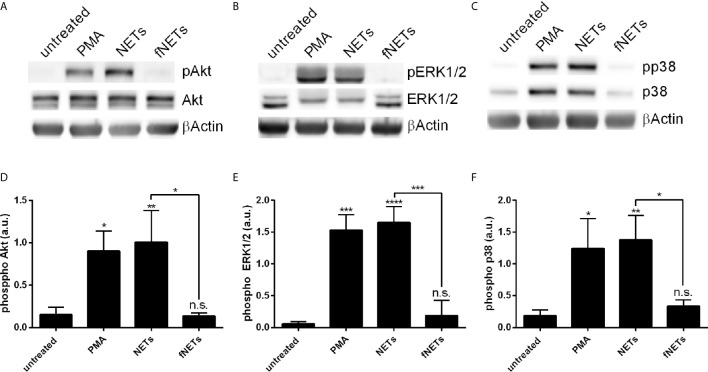
NET-induced phosphorylation of p38, Akt and ERK1/2. Neutrophils were treated with PMA, NETs, fNETs or left untreated for 15 minutes. Phosphorylation of Akt, ERK1/2 and p38 were analyzed by Western Blot. **(A–C)** Representative Western blots. **(D–F)** Phosphorylation of Akt, ERK1/2 and p38 were quantified by densitometry analysis. Signals of phosphoproteins were related to the signal of corresponding unphosphorylated proteins. Statistical analysis by ordinary one-way ANOVA with a *post hoc* Turkey’s test. Asterisks above the bars indicate significance compared to untreated cells. n=3, *=p ≤ 0,05, **=p ≤ 0,01, ***=p ≤ 0,001, ****=p ≤ 0,0001. n.s, not significant; a.u., arbitrary units corresponding to the ratio of phosphorylated to non-phosphorylated proteins.

Having seen that exposure to NETs results in the activation of several neutrophil functions we next investigated which signal pathways are engaged by NETs. Phosphorylation of Akt, ERK1/2 and p38 was analyzed, since these kinases are known to be involved in ROS production by NOX2 (Akt, ERK1/2, p38) and formation of NETs (Akt and ERK1/2) (2, 12). Neutrophils were treated with NETs and the phosphorylation of Akt, ERK1/2 and p38 was assessed by Western blot (**Figure 8**). NETs strongly induced the phosphorylation of Akt (**Figures 8A, D**), ERK1/2 (**Figures 8B, E**) and p38 (**Figures 8C, F**). fNETs had no effect on the phosphorylation of these kinases (**Figure 8**). These data indicate that NETs activate signaling pathways that include the phosphorylation of Akt, p38 and/or ERK1/2.

## Discussion

The function of neutrophil extracellular traps was initially reported to capture and kill pathogens upon infection. However, more and more evidence emerge, that NETs represent not only an effector mechanism in the defense against pathogens, but also exert a modulatory effect on cells involved in inflammatory and immune responses such as macrophages, dendritic cells and T-lymphocytes (7, 22, 25, 27, 40, 41). Surprisingly little is known about the effect of NETs on neutrophils. The effect of NETs on neutrophils, is of particular interest at sites of inflammation. Since neutrophils are the first cells to arrive at a site of inflammation and are also the most abundant leukocytes at the site of inflammation, they are most likely the first leukocytes to encounter NETs that were released during the early stage of inflammation. Therefore, activation of neutrophils by NETs have the potential to exert a strong regulatory effect on the development of inflammation.

In this study we addressed the question, whether NETs exert a proinflammatory effect on neutrophils. Our results show that NETs activate the effector functions of neutrophils including exocytosis, ROS production, NET formation and phagocytosis and also induced the secretion of the proinflammatory chemokine IL-8 and the B-cell-activating cytokine BAFF. Activation of neutrophils occurred in a concentration-dependent manner. The observed activating effects were the result of the interaction of neutrophils with NETs and not due to the presence of soluble stimuli e.g. remaining PMA or damage-associated molecular pattern (DAMPs) in the NET preparations since NETs filtered through a 0.2 µm filter (fNETs) did not activate neutrophils. Such a filtration step was shown in previous studies to sufficiently eliminate NET structures leaving all soluble factors in the NET preparation (25).

Intriguingly, digestion of NETs with DNase I did not completely abrogate but delay the activation of neutrophils by NETs (**Figure 1**). However, digestion with DNase I did not completely remove NETs, since a low amount of DNA could still be detected after 2 h of digestion (**Supplementary Figure 2**). It is tempting to speculate that the remaining DNA is associated with NET components e.g. in nucleosomes, since nucleosomes are shown to activate neutrophils in a similar manner as we observed for NETs including increased phagocytosis (36, 42), activation of ROS production (42), exocytosis and the secretion of IL-8 (36, 42). Thus, the DNA backbone of NETs might not be necessary for the general activation, but allow a better sensing of NETs by neutrophils.

Inhibition of NOX2 by VAS2870 not only abrogated ROS production by NET-exposed neutrophils (**Figures 2E, F**), but also abolished NET formation (**Figure 3E**). Therefore, we identified NETs as a strong activator of NOX2 activity and showed that NET-induced NET formation occurs by the NOX2-dependent pathway. The NET-induced NET formation suggests a self-amplifying mechanism that contributes to the potentiation of an inflammatory response. Activation of NET-induced NET formation could lead to the activation of later arriving neutrophils that in return release NETs that activate further neutrophils. Previous studies report similar mechanisms for a NET-associated feed forward loop to induce further NETs (21, 29, 30, 43). Intriguingly, Agarwal et al., 2019 (29) report, that disruption of NETs either mechanically or by DNase I digestion is required to enhance an inflammation by inducing NET-induced NET formation which they refer to as secondary NETosis. Thus, the proposed amplification mechanism might occur under certain conditions, where NETs are disrupted. In our present work vigorous pipetting may have caused mechanical disruption of NETs and thus contributing to the observed strong activating effects on neutrophil functions.

Our findings, that NET-exposure increases phagocytosis of opsonized bacteria by neutrophils (**Figure 4**) suggest that NETs are involved in both, the extracellular defense against pathogens by direct binding and killing of pathogens and also in improving the intracellular defense by increasing phagocytosis of pathogens by neutrophils and a subsequent bombardment with ROS. In fact, exposure of neutrophils to NETs that were infected with the intracellular parasite *L. donovani* increased intracellular killing of this parasite (**Figure 6**). This result is particularly interesting, since these parasites are able to surpass intracellular killing by neutrophils [reviewed in (44)]. Thus, NETs not only prevent infections with pathogens by direct interaction with the invading pathogen, but also by increasing the intracellular killing of microbial pathogens by neutrophils.

We showed that NETs are not only able to induce the release of pre-formed cytokines e.g. IL-8 (**Figure 7A**) but are also able to induce the secretion of cytokines that require *de novo* synthesis such as BAFF (**Figure 7B**). Albeit unstimulated neutrophils contain low amounts of pre-formed BAFF, they require stimulation e.g. by G-CSF to release larger amounts of BAFF (38, 45). Thus NET-exposure to neutrophils does not only induce an immediate cellular response by activating effector functions, but also has the potential to change neutrophil plasticity by inducing *de novo* synthesis of proteins. However, exposure of neutrophils to isolated NETs did not induce the secretion of TNFα (**Figure 7D**), albeit NET-induced secretion of TNFα has already been reported (22). One reason for this discrepancy could be differences in the isolation protocol, e.g. the use of DNase I and subsequent addition of EDTA, that affect the activation of neutrophils. Nevertheless, NET-induced BAFF secretion by neutrophils suggests, that NETs play an active role in shaping the adaptive immune response, since BAFF is important for the activation of B cells (46). However, excessive release of BAFF is associated with the formation of auto-antibodies (47) and an increased release of BAFF induced by NETs could further link NET release to the development of auto-immunity.

An important step in the characterization of the NET-induced activation of neutrophils is to identify the receptor engaged by NETs. Two recent studies report involvement of TLR9 (29) and TLR8 (30) in the activation of neutrophils by NETs. However, ligand binding of TLR8 or TLR9 would not activate the effector functions of neutrophils that were investigated in our study. Therefore, recognition of NETs by these receptors is unlikely the sole mechanism responsible for NET-induced activation of neutrophils. For instance, TLR8 or TLR9 activation only primes NOX2, but does not induce ROS production (30, 48, 49). However, further studies are required to identify the activating component of NETs and the receptor engaged by NETs. Our observation that NETs induce phosphorylation of p38, ERK1/2 and Akt (**Figure 8**) provides a first step to identify signal cascades induced by NET exposure and can be used as a starting point for future studies.

In conclusion, we showed that NETs activate proinflammatory functions of human neutrophils which suggests a role of NETs in a self-amplification mechanism of inflammation. Our findings further suggest that NETs actively participate in the activation of an efficient antimicrobial response by activating neutrophils. However, this NET-induced amplification of inflammatory functions may also be detrimental in chronic inflammation, auto-inflammatory and autoimmune conditions by causing tissue damage due to the increased release of ROS and promote the formation of auto-antibodies by excessive BAFF secretion.

## Data Availability Statement 

The raw data supporting the conclusions of this article will be made available by the authors, without undue reservation.

## Ethics Statement

The studies involving human participants were reviewed and approved by Ethical Committee of the University of Lübeck (20-097). The patients/participants provided their written informed consent to participate in this study.

## Author Contributions

DD and TW equally contributed to this study and designed the study, carried out experiments and wrote the manuscript. SM conducted experiments. MB and TL designed the project and wrote the manuscript. All authors contributed to the article and approved the submitted version.

## Funding

This work was carried out in the frame of the RTG1727 “Modulation of auto-immunity” and was financially funded by the *Deutsche Forschungsgemeinschaft* (DFG).

## Conflict of Interest

The authors declare that the research was conducted in the absence of any commercial or financial relationships that could be construed as a potential conflict of interest.
