# Supplementary material for: Neutrophil Extracellular Traps Activate Proinflammatory Functions of Human Neutrophils
Source: Front Immunol. 2021 Jun 8;12:636954. doi: 10.3389/fimmu.2021.636954 (PMC8217666; doi:10.3389/fimmu.2021.636954)
Supplement: Supplementary file 1 [file DataSheet_1.pdf]

Supplementary Material

Supplementary Figures

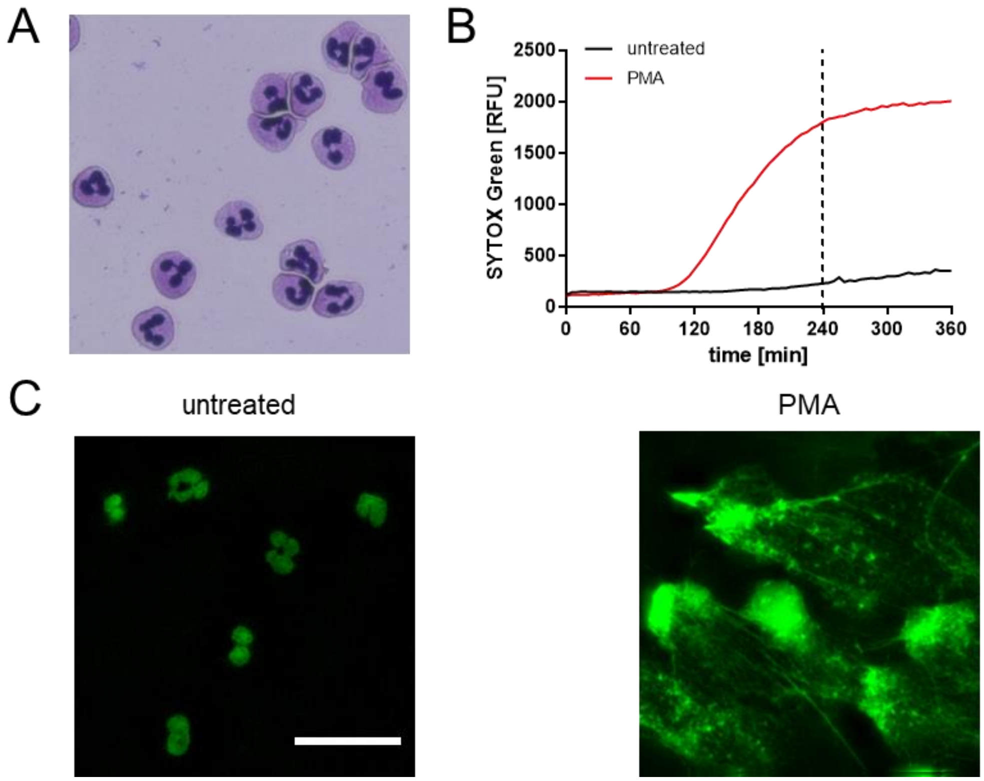

Supplementary Figure 1: Quality control of the isolated NETs. A) Freshly isolated neutrophils were stained with Giemsa to assess the purity of isolated cells **B)** A portion of neutrophils from cultures used for the NET isolation were used for a SYTOX Green assay to control NET formation. Dashed line indicates the start of the NET isolation from the culture used for the isolation. **C)** Fluorescence microscopy of neutrophils from cultures used for the NET isolation. Neutrophils were incubated for 4 h at 37°C. Released NETs were visualized by SYTOX green staining. Size bar = 20 µm.

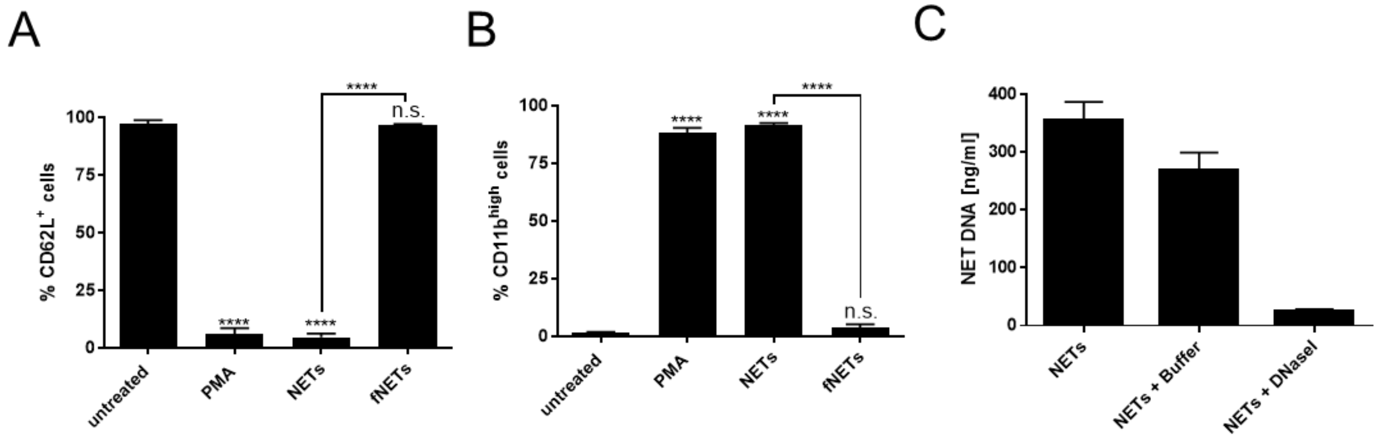

Supplementary Figure 2: Effect of NETs on cell surface activation markers of resting neutrophils. A, B) Neutrophils were cultured for 1 h in the presence of PMA, NETs, fNETs or were left untreated. The cell surface expression of CD62L **(A)** and CD11b **(B)** were assessed by flow cytometry. Statistical analysis by ordinary one-way ANOVA with a *post hoc* Turkey's test. Asterisks above the bars indicate significance compared to untreated cells. n=3, ****=p≤0,0001. n.s.=not significant. **C)** Digestion of NET DNA by DNase I was analyzed by Picogreen assay. NETs were digested with 80 U/ml DNase I for 2 h at 37°C. n=4

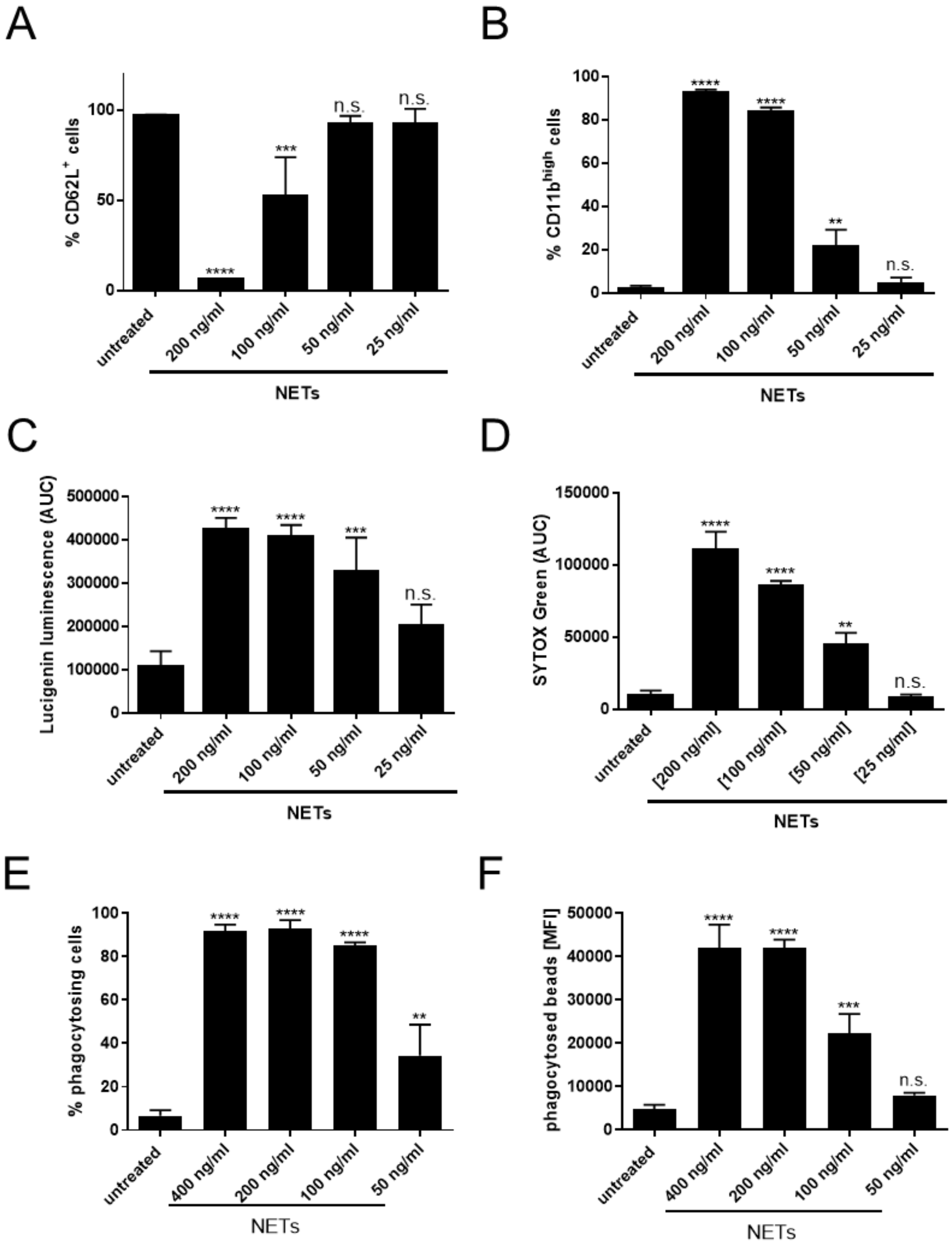

Supplementary Figure 3. Activation of neutrophils exposed to NETs occurs in a concentration-dependent manner. Neutrophils were exposed to different concentration of NET DNA and analyzed for their activation. Shedding of CD62L **(A),** expression of CD11b **(B),** extracellular ROS production **(C),** formation of NETs **(D)** and activation of phagocytosis **(E, F)**. Statistical analysis by one-way ANOVA with a post hoc Dunnett's test compared to untreated cells. n=3 **=p≤0,01 ***=p≤0,005 ****=p≤0,0001 n.s.= not significant

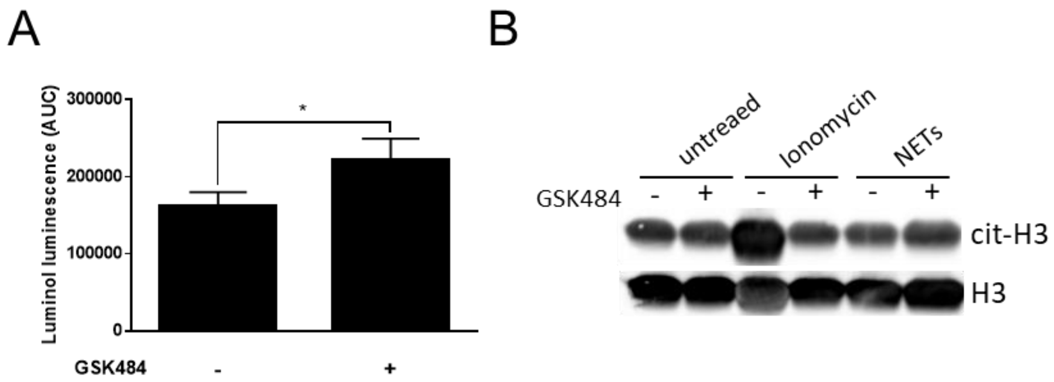

Supplementary Figure 4: Inhibition of PAD4 activity by GSK484. Neutrophils were treated with the PAD4 inhibitor GSK484 [10 µM] for 30 minutes. **A)** Cells were then exposed to NETs and the ROS production was analyzed by measuring luminol luminescence. **B)** Cells were then exposed to NETs or ionomycin for … min and the levels of citrullinated H3 as well as total H3 were assessed by western blotting. Ionomycin-stimulated neutrophils served as a positive control for enhanced PAD4 activity. Blots representative for three independent experiments.
